# Leaf Tissue‐Specific Phosphorus Allocation Is Linked to Leaf Lifespan in Chickpea Accessions

**DOI:** 10.1111/pce.70704

**Published:** 2026-06-28

**Authors:** Xiaolong Feng, Peta L. Clode, Shuyan Li, Shu Tong Liu, Jiayin Pang, Gaétan Glauser, Kadambot H. M. Siddique, Hans Lambers

**Affiliations:** ^1^ School of Biological Sciences The University of Western Australia Perth Western Australia Australia; ^2^ The UWA Institute of Agriculture The University of Western Australia Perth Western Australia Australia; ^3^ Centre for Microscopy, Characterisation and Analysis The University of Western Australia Perth Western Australia Australia; ^4^ Neuchâtel Platform of Analytical Chemistry University of Neuchâtel Neuchâtel Switzerland

**Keywords:** Jasmonic acid, leaf lifespan, leaf tissue P distribution pattern, photosynthetic P‐use efficiency, P‐remobilisation efficiency

## Abstract

Plants strategically allocate phosphorus (P) among leaf tissues to support physiological functions. However, there is limited understanding of contrasting leaf cellular P‐allocation patterns and their physiological consequences. We quantified leaf P fractions, photosynthetic P‐use efficiency (PPUE), and P‐remobilisation efficiency (PRE) in five chickpea accessions exhibiting contrasting cellular P‐allocation patterns. Leaf lifespan and concentrations of key phytohormones were also measured, including abscisic acid (ABA), salicylic acid (SA) and jasmonates. Cellular P‐allocation patterns were more strongly associated with leaf lifespan than with PPUE or PRE. Accessions allocating a greater proportion of P to the mesophyll exhibited longer leaf lifespan, which was associated with lower jasmonate concentrations, rather than ABA. Surprisingly, greater P allocation to the epidermis was driven by a higher proportion of metabolite P. At the cellular level, we primarily observed a significant negative correlation between P and calcium concentrations in mesophyll cells, but not in the epidermis. Our findings provide the first evidence that leaf tissue‐specific P‐allocation patterns vary among chickpea accessions. Preferential allocation of P to photosynthetically active cells was associated with longer leaf lifespan. Differences in jasmonate concentrations accompanied these patterns. Collectively, these findings highlight new opportunities to enhance P‐use efficiency in crop breeding programs.

## Summary statement

Chickpea accessions exhibit genotypic variation in leaf tissue‐specific phosphorus (P) allocation under moderate P availability, and this variation is associated with differences in leaf lifespan. We further provide evidence of negative correlations between cellular P and Ca concentrations in mesophyll tissues. In addition, increased epidermal P allocation is associated with higher metabolite P rather than expanded inorganic P pools.

## 1 Introduction

Phosphorus (P) is an essential macronutrient that plays a critical role in numerous physiological and biochemical processes in plants, including energy transfer (ATP), nucleic acid synthesis, photosynthesis, and membrane formation (Vance et al. 2003; Veneklaas et al. 2012). Despite its importance, P is limiting in more than 30% of agricultural systems (MacDonald et al. 2011; Solangi et al. 2023). Approximately 30%–65% of total soil P exists in organic forms that must first be mineralised into inorganic phosphate (P_i_) through microbial activity or root‐secreted enzyme processes before becoming available for plant uptake. Moreover, even available P_i_ typically occurs at very low concentrations (μM range), due to its strong sorption to aluminium and iron oxides and hydroxides under acidic conditions (pH < 7) and to calcium under alkaline conditions (pH > 7) (Lambers 2022; Islam et al. 2024). As concerns grow regarding sustainable agriculture and the depletion of finite phosphate rock reserves, improving the understanding of plant P acquisition and utilisation has become increasingly important.

Leaves, as the primary sites of gas exchange and photosynthesis, typically contain higher P concentration than roots and stems (Freschet et al. 2010; Feng et al. 2023). Two key P‐use strategies have therefore been widely investigated: photosynthetic P‐use efficiency (PPUE), defined as the amount of carbon assimilated per unit of leaf P (Wen et al. 2023; Shen et al. 2024; Gille et al. 2024), and P‐remobilisation efficiency (PRE), defined as the plant's ability to resorb P from senescing leaves before abscission (Veneklaas et al. 2012). In addition, the allocation of P fractions within leaves provides an integrated perspective on internal P utilisation strategies. For example, increased allocation to metabolite P (e.g., enzyme substrates) is associated with higher PPUE in Proteaceae species growing in P‐impoverished regions of south‐western Australia (Shen et al. 2024; Gille et al. 2024). However, how these strategies relate to tissue‐specific P allocation within leaves remains unclear.

Leaves contain specialised tissues, including the epidermis, palisade mesophyll, spongy mesophyll, and vascular bundles, each performing distinct physiological functions. The epidermis protects internal tissues from mechanical damage and pathogen invasion, whereas chloroplast‐rich mesophyll cells capture light and fix carbon via the Calvin cycle (Evans 1999). Different cell types may preferentially accumulate specific elements for their physiological roles or contributions to stress tolerance. For example, mungbean (*Vigna radiata* L.) cv. Jade AU maintains lower Na and Cl concentrations in chloroplasts and preferentially accumulates K in mesophyll cells compared with cv. BARI Mung‐6, contributing to higher photosynthetic efficiency and improved salt tolerance (Iqbal et al. 2024). In addition, some elements may interact antagonistically. P and calcium (Ca) are typically compartmentalised into different cells or organelles (Boursier and Läuchli 1989; Ding et al. 2018), or sequestered in inert crystal‐like forms (Paiva 2019). When co‐localised, their concentrations must be tightly regulated to remain below precipitation thresholds (Karley et al. 2000; Conn and Gilliham 2010). Whether the relationship between these two elements varies among cell types remains unknown.

Current understanding of the mechanisms governing tissue‐specific P allocation and its physiological consequences remains limited. Conn and Gilliham (2010) proposed a monocot–dicot model of tissue P distribution, suggesting that monocots preferentially allocate P to mesophyll cells, whereas dicots tend to allocate more P to the epidermis. Although this model has been supported in some species (Karley et al. 2000; Conn and Gilliham 2010), including barley (*Hordeum vulgare* L.) (Fricke et al. 1994) and lupin (Ding et al. 2018), several native dicot species from P‐impoverished soils in Western Australia preferentially allocate P to mesophyll cells (Guilherme Pereira et al. 2018; Hayes et al. 2018). These observations challenge the general model and suggest that additional mechanisms may regulate tissue‐specific P allocation. For example, Proteaceae species from P‐impoverished habitats preferentially allocate P to mesophyll cells, which increases PPUE (Hayes et al. 2018). In contrast, in chickpea, increased PPUE under low‐P conditions is associated with greater allocation to nucleic acid P (primarily ribosomal RNA), rather than with cell‐specific P allocation (Wen et al. 2023). Nevertheless, tissue‐specific P allocation may enhance PRE, as concentrating P within specific cell types could facilitate localised expression of phosphatase enzymes (Hayes et al. 2018; Guilherme Pereira et al. 2019). These enzymes convert organic P into inorganic forms, enabling P remobilisation via phloem loading (Veneklaas et al. 2012; Stigter and Plaxton 2015). However, empirical evidence regarding the effects of cell‐specific P allocation on PRE remains limited and requires further investigation.

Tissue‐specific P allocation may also influence leaf lifespan. During leaf senescence, up‐regulation of some hormones can trigger programmed cell death (Hu et al. 2017; Xu et al. 2025), accompanied by the remobilisation of nutrients, including nitrogen (N), P, and potassium (K). Since chloroplasts are among the first organelles to undergo degradation during senescence, and mesophyll cells typically senesce earlier than epidermal cells (Lim et al. 2007; Schippers et al. 2015), greater P allocation to mesophyll cells— which are located closer to vascular tissues—may reduce the distance required for nutrient export. This proximity may reduce the synthesis of senescence‐promoting hormones, such as abscisic acid (ABA) and jasmonates (He et al. 2002; Lee et al. 2015), thereby potentially affecting leaf lifespan.

To better understand leaf cell‐specific P‐allocation patterns and their physiological implications, it is important to integrate cell‐specific P allocation, P fractions, phytohormone profiles, and key physiological traits such as PPUE and PRE. Based on preliminary experiments (Feng et al. 2026), we selected five chickpea accessions with contrasting leaf PRE. We hypothesised that: (1) preferential allocation of P to mesophyll is associated with higher PRE; (2) increased allocation of P to mesophyll is associated with longer leaf lifespan, and with changes in phytohormone concentrations; (3) increased P allocation to epidermal cells is associated with a greater proportion of inorganic phosphate (P_i_), potentially reflecting a storage role of the epidermis; and (4) the correlation between the concentrations of P and Ca differs among leaf tissues.

## 2 Materials and Methods

### 2.1 Plant Material and Growth Conditions

Five chickpea accessions were selected for this study based on a preliminary experiment with 266 accessions grown to physiological maturity, which revealed substantial genotypic variation in PRE under moderate P supply (Feng et al. 2026). Two accessions exhibited high PRE, and three exhibited low PRE. Details on the origin and market type are available in Table S1.

Plants were grown in 2 L black pots (49 pots per accession; 245 pots total) to provide sufficient individuals for the measurement of multiple traits. The experiment was conducted from May to December 2024 in a temperature‐controlled glasshouse at The University of Western Australia, Perth (31°58′ S, 115°49′ E). The average daily temperature was 22/15°C (day/night), with a mean relative humidity of 75%. Pots were periodically rearranged to minimise spatial effects.

A mixture of field soil and sterilised washed river sand (1:4, w:w) was used as the growth medium. Field soil, containing 20 µg total plant‐available P per g, was collected from the upper 0.15 m at Cunderdin Agriculture College, 158 km east of Perth. Each pot contained 2.5 kg of the air‐dried soil mixture. Exogenous P was supplied as KH_2_PO_4_ to achieve a final soil P concentration of 25 mg P kg^−1^. Basal nutrients included 15 mg N kg^–1^ as Ca(NO_3_)_2_·4H_2_O, 3.75 mg N kg^–1^ as NH_4_Cl, 50 mg S kg^–1^ as K_2_SO_4_, 0.12 mg B kg^−1^ soil as H_3_BO_3_, 2 mg Zn kg^–1^ as ZnSO_4_·7H_2_O, 0.5 mg Cu g^–1^ as CuSO_4_·5H_2_O, 0.4 mg Mo kg^–1^ as Na_2_MoO_4_·2H_2_O and 5 mg Fe kg^–1^ as FeNaEDTA (Wen et al. 2023). An additional 5 mg N kg^–1^ was applied 1 month after sowing as Ca(NO_3_)_2_·4H_2_O.

Before sowing, seeds were treated with fungicide (P Pickel‐T®, 360 g L^−1^ thiram, 200 g L^−1^ thiabendazole) to prevent early fungal infection. Two seeds were sown per pot at a depth of 2.5 cm, and thinned to one plant 15 days post‐sowing. Peat‐based chickpea rhizobium inoculum was applied to the planting holes and soil surface 2 weeks after planting. After establishment, the soil surface was covered with plastic beads to minimise evaporation. Soil water content (SWC) was maintained at 80% of pot capacity, measured by weighing every other day. Following pod development, SWC was maintained at 70% until harvest.

### 2.2 Photosynthetic Traits and Leaf Lifespan

Six weeks after sowing, plants under moderate P availability exhibited strong main—stem dominance. Photosynthetic measurements were conducted on young, fully expanded leaves of the main stem. Upon full expansion of the 13th leaf, leaves were tagged for identification. Gas exchange measurements of the 15th leaf were performed using a LI‐6800 Portable Photosynthesis System (LI‐COR, Lincoln, NE, USA) between 9:30 and 12:00 local time. Conditions in the leaf chamber were maintained as follows: 400 μmol mol^–1^ CO_2_ concentration, 1,500 μmol photons m^–2^ s^–1^ photosynthetically active radiation using a 90% red LED light source, ~60% relative humidity, 25°C temperature, 500 μmol s^–1^ flow rate, and a mixing fan at 10,000 rpm to ensure rapid homogenization of CO_2_ and H_2_O vapour. The following morning, the 15th leaf and the adjacent 14th leaf were harvested pre‐dawn, placed in an ice box, and transported to the laboratory. Leaves were weighed, for fresh weight, and scanned at 300 dpi using a flatbed scanner (Epson Perfection v850 Pro, Tokyo, Japan). Leaf area was analysed with WinRhizo 2009 software (Regent Instruments Inc., Quebec, Canada). Leaves were then oven‐dried at 60°C for 48 h to determine dry weight, and leaf mass per area (LMA) was calculated as the ratio of leaf dry weight to leaf area.

For pigment analysis, 3–4 leaflets from the 15th leaf of other individuals were collected into plastic bags, placed on ice, and weighed immediately upon return to the laboratory. The samples were then immersed in 10 mL of 80% (v/v) acetone and wrapped in aluminium foil for 48 h. Chlorophyll and carotenoid concentrations were determined spectrophotometrically at 663, 645, and 470 nm (Thermo Fisher Scientific, AQ8100, Waltham, MA, USA) following Chazaux et al. (2022).

Leaf death was recorded when the 15th leaf was fully senesced and detached upon a gentle touch. For ICC7184, leaves did not shed after full senescence; in this case, the time when the leaf became fully yellow‐brown was recorded as death. Leaf lifespan was defined as the interval between full expansion and the time of leaf death.

### 2.3 Leaf Tissue‐Specific Element Analyses

Leaf subsamples for cellular‐scale analysis were collected from the most recently fully expanded leaves (the 15th leaf, same as used for photosynthesis measurements), following the protocols described by Hayes et al. (2018) and Guilherme Pereira et al. (2018). Briefly, ~2 × 3 mm fragments were excised from the middle region of each half‑leaflet, on both sides of the midrib. Two fragments were taken from two separate leaflets per plant. Each fragment was immediately mounted on an aluminium pin using optimal cutting temperature compound (Tissue‑Tek, Torrance, CA, USA) and rapidly frozen in liquid nitrogen. Samples were transported to the laboratory in a liquid nitrogen dewar and stored until analysis. Rapid freezing preserves the spatial distribution of elements, making it suitable for cellular‐scale quantification (Ding et al. 2018; Guilherme Pereira et al. 2018; Wen et al. 2023; Iqbal et al. 2024).

Frozen samples were prepared using a cryo‐microtome (Ultracut EM UC7 with FC7 cryo‑chamber, Leica Microsystems GmbH, Vienna, Austria) at −120°C to obtain flat, clean cross‐sections. Rough planing was performed using a glass knife at 1 µm, 750 nm, and 500 nm steps, followed by fine sectioning at 250 nm and 100 nm.

Cryoplaned samples were transferred directly from the cryo‐microtome to a high‑vacuum sputter‐coating system (ACE600, Leica Microsystems GmbH, Vienna, Austria) and coated with 20 nm chromium. Coated samples were then transferred, under vacuum, to a field emission scanning electron microscope (cryo‑SEM, JSM‐IT800HL FESEM, JEOL Tokyo, Japan) equipped with a Leica VCT500 cryostage and an Oxford Ultim‐Max 170 mm^2^ X‑ray detector coupled to AZtec software (Oxford Instruments, Abingdon, UK). Beam currents were measured with an in‐column probe current detector, and the instrument was calibrated before each experiment using a pure copper standard.

Elemental mapping was performed at −160°C using a 15 kV accelerating voltage and a beam current of 1.0 nA. Maps were acquired at 512‐pixel resolution over > 3,000 frames, with a dwell time of 10 µs per pixel. Drift and pulse‑pile‑up corrections were applied. After mapping, EDS data were processed using AZtec software. Different cell types, including upper and lower epidermis and the palisade and spongy mesophyll, were determined and elemental concentrations were quantified based on accumulated signal intensities from each region of interest (Table S2). Parameter details are provided in Iqbal et al. (2024). Three biological replicates were analysed, each comprising one or two mapped regions. Leaf thickness was measured from micrographs using ImageJ software. Five cross‐sections per leaflet were analysed, with three biological replicates.

### 2.4 Leaf Phosphorus Fraction

For foliar P concentration and fractionation analysis, additional subsamples were collected from young fully expanded leaves (leaves 14 and 15). Leaves from three pots were pooled to form one biological replicate. All leaf material was immediately wrapped in aluminium foil, flash‑frozen in liquid nitrogen, and stored at −80°C until freeze‑drying.

Samples were freeze‐dried for 6 days (Eyela FDU‐2100, Tokyo Rikakikai Co. Ltd, Tokyo, Japan) and ground into fine powder using a Geno/Grinder 2000 (Spex SamplePrep, Metuchen, NJ, USA). Approximately 50 mg subsample was digested in HNO_3_:HClO_4_ (3:1, v/v) and analysed by inductively coupled plasma optical emission spectroscopy (ICP‐OES, Model 5300DV, Perkin Elmer, Shelton, CT, USA) to determine concentrations of P, potassium (K), Ca, iron (Fe), manganese (Mn), and zinc (Zn). A second subsample (~100 mg) was used for P fractionation following Hayes et al. (2022).

### 2.5 Phytohormone Measurement

Phytohormones—including ABA, salicylic acid (SA), jasmonic acid (JA), jasmonoyl‐isoleucine (JA‐Ile), and 12‐oxo‐phytodienoic acid (OPDA)—were quantified in senescing leaves. When chlorophyll in the 15th leaf declined by ~50% relative to fully expanded young leaves (Figure S4), both the 15th and 14th leaves were rapidly collected, wrapped in aluminium foil, flash‐frozen in liquid nitrogen, and stored at−80°C. Freeze‐dried samples were ground into fine powder, and ~20 mg was used for hormone quantification via ultra‐high‐performance liquid chromatography coupled with tandem mass spectrometry (UHPLC–MS/MS) using an Acquity UPLC I‐Class system (Waters Corp., Milford, MA, USA) connected to a QTRAP 6500+ mass spectrometer (SCIEX, Framingham, MA, USA). Hormone extraction and quantification followed Glauser et al. (2014). Briefly, we extracted ~20 mg of freeze‐dried, finely ground leaf material showing approximately 50% chlorophyll reduction with 1 mL of ethyl acetate containing 0.5% (v/v) formic acid. Isotopically labelled internal standards (d5‐JA, d6‐SA, and 13C6‐JA‐Ile; 100 ng mL^–1^) were added to each sample prior to extraction. Samples were homogenised using a bead‐based mechanical disruption system and centrifuged at 14,000 g for 4 min. The extraction procedure was carried out twice, and the combined supernatants were evaporated under reduced pressure at 35°C. Dried extracts were re‐dissolved in 50% (v/v) methanol, centrifuged, and transferred to autosampler vials for UHPLC–MS/MS analysis. Chromatographic separation was performed using an Acquity UPLC BEH C18 column (50 × 2.1 mm, 1.7 μm particle size; Waters Corp., Milford, MA, USA). A binary mobile phase consisting of water and acetonitrile, each containing 0.05% (v/v) formic acid, was applied under gradient elution conditions. A 2‐μL aliquot of each sample was injected for analysis (Gille et al. 2024).

### 2.6 Phosphorus‐Remobilisation Efficiency

Fully senesced leaves (14th and 15th) were collected, immediately stored at −80°C, and freeze‐dried. The P concentration in these leaves (P_s_) was determined as described above. PRE was calculated:

$$\text{PRE}\;(\%)=(P_{g}-P_{s})/P_{g}\times 100$$

where P_g_ represents the P concentration in the corresponding young fully‐expanded leaves.

### 2.7 Statistical Analyses

Differences in leaf cellular nutrient concentrations were assessed using linear mixed‐effects models, with individual plants included as a random effect (Ding et al. 2018; Wen et al. 2023). Pairwise comparisons were performed using Tukey‐Kramer‐adjusted estimated marginal means (‘emmeans’ package). Residual plots were visually inspected for heteroscedasticity. In the presence of heteroscedasticity, appropriate variance structures were incorporated if they significantly improved model fit, as assessed by the Akaike information criterion (Zuur et al. 2009). One‐way analysis of variance was used to examine differences in elemental concentrations and physiological traits among accessions with post‐hoc pairwise comparisons using Tukey's HSD test. Normality of residuals was assessed using the Shapiro‐Wilk test and visual inspection of Q‐Q plots; homogeneity of variance was evaluated using Levene's test. Differences in phytohormone concentrations among accessions were assessed using a non‐parametric multiple comparison approach, with pairwise comparisons performed using Dunn's test and Benjamini–Hochberg (BH) correction for multiple testing. All statistical analyses and figures were generated in R 4.3.0 (R Core Team, 2023).

## 3 Results

### 3.1 Variation in Total Foliar and Cellular Element Concentrations

No significant differences in total foliar P concentration were observed among the five chickpea accessions (*F*_*4,15*_ = 2.09, *p* = 0.13).

The highest mean foliar P concentration was measured in ICC7323 (2.44 mg g^–1^), while ICC9590 had the lowest (2.16 mg g^–1^). In contrast, total foliar N concentration varied significantly (*F*_*4,15*_ = 3.38, *p* = 0.04), with ICC10885 exhibiting lower N (39.1 mg g^–1^) than ICC7323 (44.4 mg g^–1^), whereas the other accession did not differ significantly. Significant genotypic differences were also observed in leaf Ca concentration, ranging from 26.7 mg g^–1^ in ICC13219 to 15.1 mg g^–1^ in ICC10885 (Table 1). Leaf N:P ratios varied little among chickpea accessions, ranging from 17.0 ± 0.6 in ICC10885 to 19.3 ± 0.4 in ICC9590, and did not differ significantly among accessions (*F*_*4,15*_ = 1.72, *p* = 0.20).

**Table 1:** Nitrogen (N_g_), calcium (Ca), and phosphorus (P_g_) concentrations in young fully‐expanded leaves of five chickpea accessions. Data are presented as means ± SE (*n* = 4). Different letters indicate significant differences among accessions based on Tukey's HSD tests (*p* < 0.05). No letters are shown for P_g_ because no significant differences among accessions were detected.

	ICC 13219	ICC 7184	ICC 9590	ICC 7323	ICC 10885
P_g_ (mg g^–1^)	2.43 ± 0.09	2.32 ± 0.07	2.16 ± 0.04	2.44 ± 0.10	2.29 ± 0.08
Ca (mg g^–1^)	26.7 ± 0.4^a^	21.6 ± 0.7^b^	19.9 ± 0.8^bc^	17.4 ± 1.0 ^cd^	15.1 ± 0.8 ^d^
N_g_ (mg g^–1^)	44.0 ± 1.0^ab^	41.9 ± 0.3^ab^	41.7 ± 0.2^ab^	44.4 ± 1.2^a^	39.1 ± 2.0^b^

At the tissue level, cellular P concentrations varied markedly among accessions and cell types. In ICC7184, P concentration was higher in epidermal than mesophyll cells, with the upper epidermis showing an exceptionally high estimated marginal mean of 81 µmol g^–1^. In contrast, the corresponding value in ICC13219 was only 8 µmol g^–1^ (Figure 1). In the remaining accessions, mesophyll P concentrations ([P]) generally exceeded those in the epidermis. Epidermal [P] differed substantially among accessions, being lowest in ICC13219 (8 µmol g^–1^), intermediate in ICC7323 and ICC10885 (15 and 13 µmol g^−1^, respectively), and the highest in ICC7184 (57 µmol g^–1^; Figure 1). Mesophyll [P] showed a narrower range, with ICC10885 exhibiting the lowest mean (24 µmol g^–1^) and all other accessions exceeding 25 µmol g^−1^, reaching a maximum in ICC13219 (28 µmol g^–1^).

**Figure 1:**
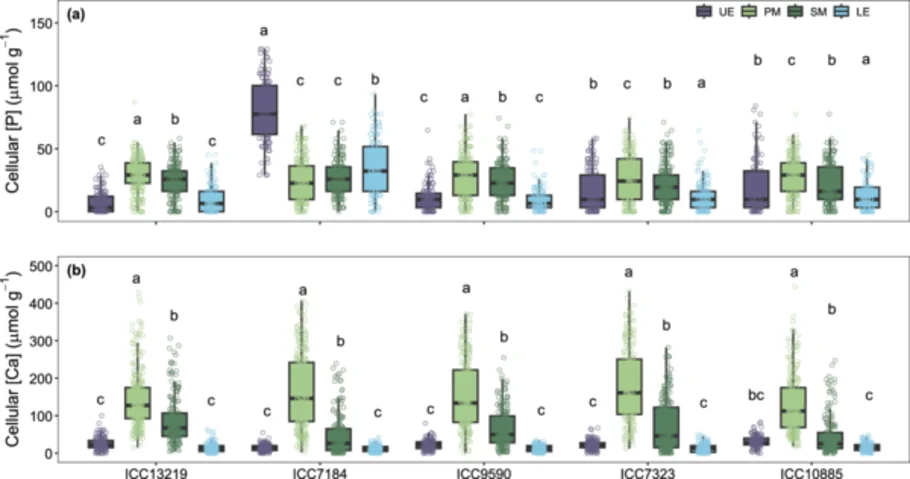
Cellular concentrations of phosphorus ([P]) and calcium ([Ca]) in five chickpea accessions supplied with 25 µg Pg^−1^ soil grown in a glasshouse. (a) Cellular [P] (μmol P g^−1^) and (b) [Ca] (μmol Ca g^−1^) in upper epidermis (UE), palisade mesophyll (PM), spongy mesophyll (SM), and lower epidermis (LE). Each point represents a cell‐level measurement obtained by cryo–scanning electron microscopy X‐ray microanalysis. Box plots show the median, 25th and 75th percentiles, with whiskers extending to 1.5 x the interquartile range. Measurements were taken from three leaflets biological replicate. Different letters within each accession indicate significant differences among cell types (*p* < 0.05), determined using Tukey–Kramer post‐hoc tests on linear mixed‐effects models, with individual plants included as a random effect.

Compared with [P], variation in cellular Ca concentrations was smaller (Figure 1, Figure S1). Across all accessions, Ca consistently accumulated more in mesophyll than epidermal cells (Figure 1). Despite this conserved pattern, Ca concentrations differed significantly among accessions. Epidermal Ca was lowest in ICC7184 (13 µmol g^–1^) and highest in ICC10885 (23 µmol g^–1^). In mesophyll cells, ICC10885 exhibited the lowest Ca concentration (87 µmol g^−1^), significantly lower than in the other accessions, which ranged from 115 µmol g^–1^ in ICC9590 to 124 µmol g^–1^ in ICC7323, with no significant differences among them.

### 3.2 Correlations Between Concentrations of P and Ca Among Cell Types

Cellular P and Ca concentrations showed cell type– and accession‐specific patterns (Figure 2). In palisade mesophyll, the concentration of Ca was consistently negatively correlated with that of P across all accessions, with relatively strong correlations (marginal *R^2^* = 0.22–0.45, *p* < 0.001). In the spongy mesophyll, similar but weaker negative correlations were observed in four accessions (marginal *R^2^* = 0.04–0.22), whereas no significant correlation was detected in ICC10885.

**Figure 2:**
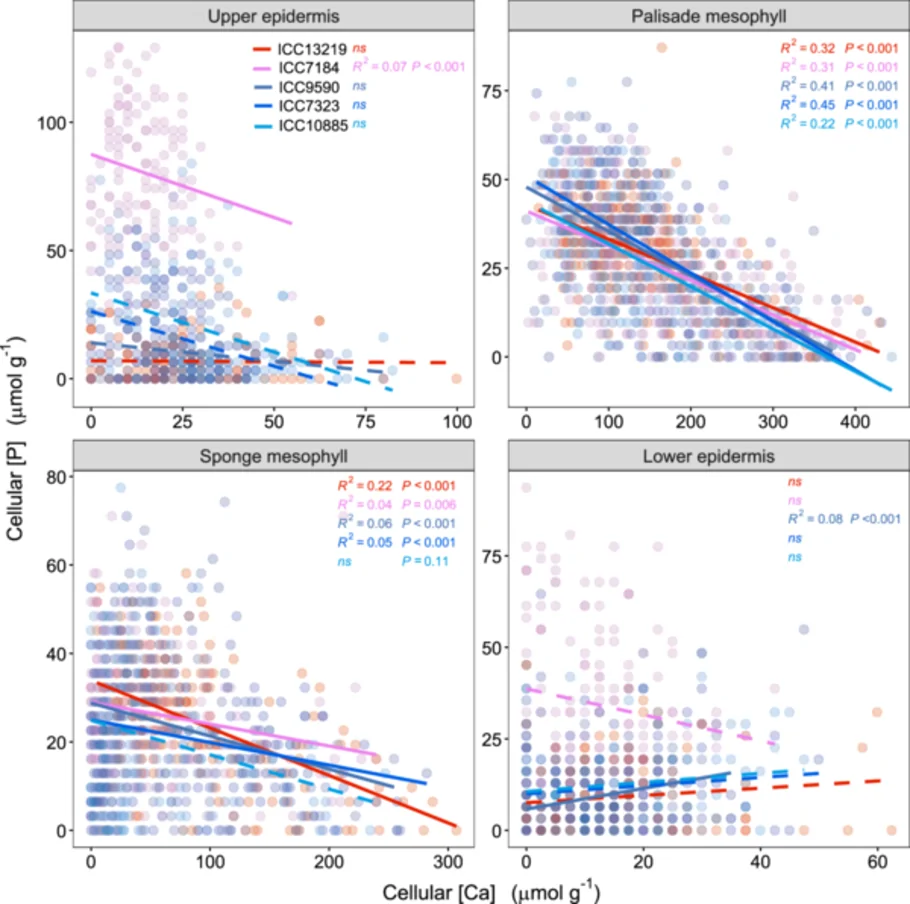
Correlations between cell phosphorus ([P]) and calcium ([Ca]) concentrations in different leaf cellular types in five chickpea accessions supplied with 25 µg P g^−1^ soil, grown in a glasshouse. Scatterplots illustrate correlations between cellular [P] and cellular [Ca] in (top left) upper epidermis, (top right) palisade mesophyll, (bottom left) spongy mesophyll and (bottom right) lower epidermis. Each point represents a cell‐level measurement by cryo‐scanning electron microscopy X‐ray microanalysis, coloured by accession; each accession had three biological replicates. Fitted lines were obtained using linear mixed‐effects models with individual plants as a random effect. Solid lines indicate significant correlations, while dashed lines indicate non‐significant correlations. Marginal *R^2^* values are shown; non‐significant results (*p* > 0.05, ns) are not displayed. [Color figure can be viewed at wileyonlinelibrary.com]

In contrast, correlations between P and Ca concentrations in epidermal cells were weaker and more variable. In the upper epidermis, a significant negative correlation was observed only in ICC7184 (marginal *R^2^* = 0.07, *p* < 0.001), whereas the other accessions showed no significant correlations. In the lower epidermis, P and Ca were largely uncoupled, with only ICC9590 showing a weak but significant positive correlation (marginal *R^2^* = 0.08, *p* < 0.001).

### 3.3 Variation in Leaf Structure, Photosynthesis and Phosphorus‐Use Efficiency

Leaf structural and physiological traits varied among chickpea accessions under moderate P supply (Figure 3). LMA, area‐based photosynthesis (A_area) and photosynthetic P‐use efficiency (PPUE) did not significantly differ among accessions (Figure 3a–c).

**Figure 3:**
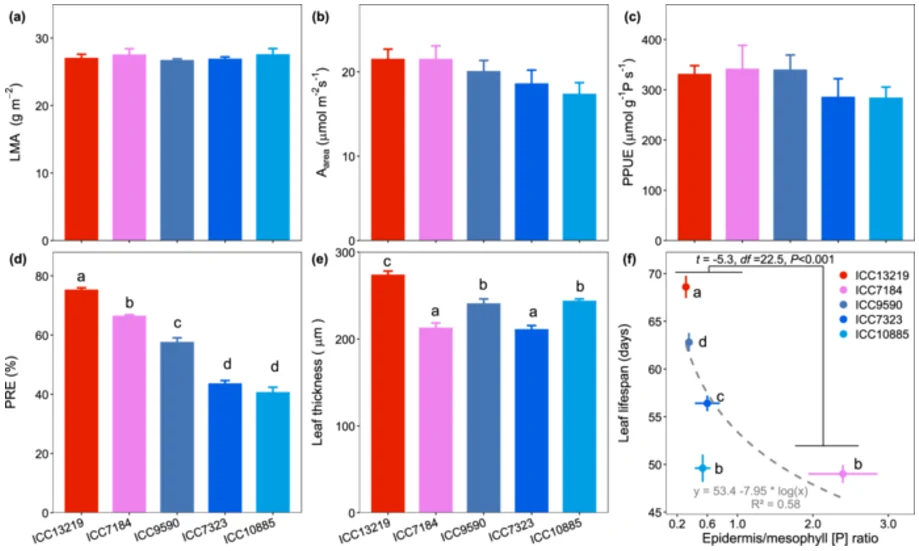
Leaf structural physiological traits in five chickpea accessions supplied with 25 µg phosphorus (P) g^−1^ soil, grown in a glasshouse. (a) Leaf mass per area (LMA), (b) area‐based photosynthesis (A_area_), (c) photosynthetic P‐use efficiency (PPUE), (d) P‐remobilisation efficiency (PRE) and (e) leaf thickness in five accessions. (f) Correlation between the ratio of epidermis‐to‐mesophyll P concentration and leaf lifespan. Data represent means ± SE (*n* ≥ 3). Different letters above bars indicate significant differences among accessions (Tukey's HSD test, *p* < 0.05). Panel (f) shows the fitted curve from on a non‐linear regression, with equation and *R^2^* displayed. Letters above points in (f) indicate significant differences in leaf lifespan, and Welch's *t*‐test was used to compare ICC7184 with other accessions. [Color figure can be viewed at wileyonlinelibrary.com]

In contrast, PRE varied markedly. ICC13219 exhibited the highest PRE, followed by ICC7184 and ICC9590, while ICC7323 and ICC10885 showed significantly lower values (Figure 3d). Leaf thickness also differed significantly: ICC13219 had the thickest leaves, ICC7184 and ICC7323 the thinnest, and ICC9590 and ICC10885 were intermediate (Figure 3e).

Leaf lifespan differed significantly among accessions and was negatively associated with the epidermis‐to‐mesophyll P concentration ratio. The estimated slope was −7.95 ± 3.94 SE, explaining 58% of the variance in leaf lifespan (*R^2^* = 0.58). Although the effect was not statistically significant (*F*_*1,3*_ = 4.07, *p* = 0.14), likely due to the small sample size (*n* = 5), the negative slope and 95% confidence interval (CI: −20.50 to 4.59), suggest a strong trend. The root mean square error was 4.93, corresponding to 8.61% of the mean leaf lifespan, indicating a reasonable model fit. Among accessions, ICC13219—with the lowest epidermal [P] — had the longest leaf lifespan, whereas ICC7323 and ICC10885 had significantly shorter lifespans. ICC7184, which had the highest epidermal [P], exhibited the shortest lifespan, as confirmed by Welch's *t*‐test (Figure 3f).

### 3.4 Differences in Leaf P Fractions

Chickpea accessions exhibited significant variation in both concentration and relative proportion of leaf P fractions, despite no significant differences in total foliar P (Figure 4). Nucleic P was the most abundant fraction, ranging from 38% in ICC13219% to 44% in ICC7323. Inorganic P accounted for 28% in ICC7184% to 34% in ICC9590, while lipid P ranged from 15% in ICC7184% to 17% in ICC9590. Metabolite P showed notable variation among accessions, with ICC7184 having the highest proportion (12%) and ICC13219 at 10%. The remaining contained less than 8% metabolite P, with ICC9590 exhibiting the lowest proportion at 6%. Residual P was the least abundant and least variable fraction, ranging from 3.0% in ICC7323% to 4% in ICC9590.

**Figure 4:**
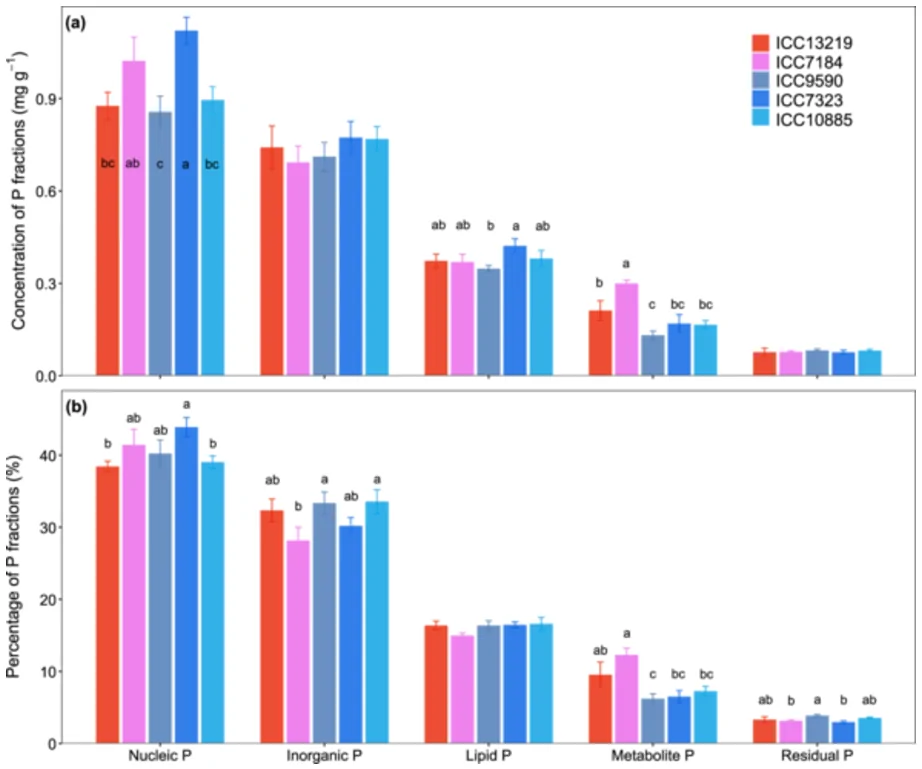
Phosphorus (P) fractions in five chickpea accessions supplied with 25 µg P g^−1^ soil, grown in a glasshouse. (a) Concentration (mg g^−1^ DW) and (b) percentage of total P. Bars represent the means ± SE (*n* = 4). Different letters above bars within each P fraction indicate significant differences among accessions, determined using the least significant difference (LSD) test. [Color figure can be viewed at wileyonlinelibrary.com]

### 3.5 Phytohormones

Significant differences in phytohormone concentrations were observed among the three evaluated accessions (ICC13219, ICC7184, and ICC10885; Figure 5). Salicylic acid concentrations varied markedly, with ICC7184 showing the highest concentration, significantly exceeding those in ICC10885 (Figure 5a). Abscisic acid concentrations differed less substantially (Figure 5b); ICC7184 tended to have a lower ABA concentration than the other accessions did, but this difference was not significant.

**Figure 5:**
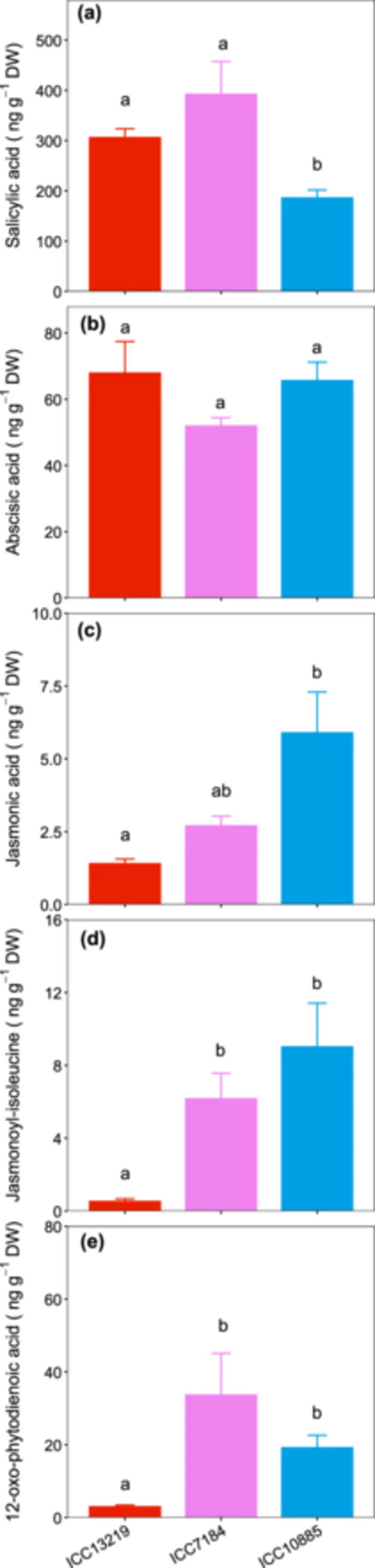
Phytohormone concentrations in leaves showing approximately 50% chlorophyll reduction from three chickpea accessions (ICC13219, ICC7184, and ICC10885) supplied with 25 µg phosphorus (P) g^−1^ soil and grown in a glasshouse. Concentrations of (a) salicylic acid, (b) abscisic acid, (c) jasmonic acid, (d) jasmonoyl‐isoleucine, and (e) 12‐oxo‐phytodienoic acid in freeze‐dried leaves are shown. Data represent means ± SE (*n* = 5). Different letters indicate significant differences among accessions, determined by Dunn's post‐hoc test with Benjamini–Hochberg (BH) adjustment (*p* < 0.05). [Color figure can be viewed at wileyonlinelibrary.com]

In contrast, JA concentrations showed pronounced differences (Figure 5c). ICC7184 had a higher JA concentration than ICC13219 did, while ICC10885 exhibited the highest JA concentration, significantly greater than that in ICC13219. A similar pattern was observed for the concentrations of jasmonoyl‐isoleucine (JA‐Ile; Figure 5d), with ICC7184 and ICC10885 showing substantially higher concentrations than ICC13219 did, and ICC10885 having the highest concentrations overall. Concentrations of OPDA, a JA precursor, also differed significantly among accessions, with ICC7184 exhibiting the highest OPDA concentration, significantly exceeding that of ICC13219 (Figure 5e). Spearman's rank correlation revealed significant negative correlations between leaf lifespan and the concentrations of JA‐related compounds, including those of JA, JA‐Ile, and OPDA (Figure S5), whereas we detected no significant correlations for the concentrations of other phytohormones.

## 4 Discussion

The mechanisms governing leaf cellular P allocation and its physiological consequences remain poorly understood (Conn and Gilliham 2010; Hayes et al. 2018). In this study, we provide the first evidence that chickpea accessions differ significantly in leaf cellular P allocation under moderate P availability. Importantly, cellular P allocation was linked to leaf lifespan: accessions allocating more P to mesophyll cells had a lower concentration of jasmonate, and exhibited the longest leaf lifespan. We also identified, for the first time, a clear antagonistic relationship between the concentrations of P and Ca in mesophyll cells, which was absent in the epidermis. Notably, our findings suggest that jasmonates, rather than the traditionally emphasised ABA (Plaxton and Lambers 2015; Xu et al. 2025), may be more closely associated with variation in leaf lifespan among chickpea accessions. Collectively, these results advance our understanding of cellular P distribution and its physiological implications in dicot crops.

### 4.1 Inconsistent Genotypic Cellular Phosphorus Allocation

Traditionally, the monocot–dicot model of leaf P distribution posits that monocots preferentially allocate P to mesophyll, while dicots allocate more to epidermal cells (Conn and Gilliham 2010; Guilherme Pereira et al. 2018; Hayes et al. 2018). However, our results challenge this observation. We observed substantial variation in leaf cellular P distribution among five chickpea accessions grown under identical moderate P supply (Figure 1), demonstrating that the model does not universally apply. Leaf N:P ratios did not differ significantly among accessions, suggesting that variation in N:P stoichiometry was unlikely to account for the observed differences in tissue‐specific P allocation and associated traits. Whether these patterns are primarily driven by genetic factors or environmental conditions, such as soil P availability, remains unclear. For example, Proteaceae native to P‐impoverished soils in Western Australia preferentially allocate P to photosynthetically active mesophyll, whereas species within the same family from P‐rich soils in Chile do not (Hawkins et al. 2008; Hayes et al. 2018). Similarly, *Hakea prostrata* maintains mesophyll P allocation even under P‐toxic conditions, while lupins consistently allocate more P to the epidermis regardless of external P supply (Ding et al. 2018; Shane 2004; Treeby et al. 1987). These examples collectively suggest that leaf cellular P distribution is predominantly genetically regulated rather than environmentally determined. Our findings support the hypothesis that cellular allocation in chickpea is under genetic control. However, the potential modulation of this trait by soil P availability remains unresolved. Further experiments across a gradient of P conditions will be necessary to clarify the environmental sensitivity of cellular P allocation in chickpea.

### 4.2 Cellular P Allocation Is More Strongly Associated with Leaf Lifespan Than Photosynthetic P‐Use Efficiency or P‐Remobilisation Efficiency

In this study, ICC7184 allocated a higher proportion of P to the epidermis and exhibited higher PRE than the other accessions, which preferentially allocated P to mesophyll cells (Figure 1). This finding contradicts our first hypothesis that preferential P allocation to the mesophyll enhances PRE. Interestingly, ICC13219 allocated the least P to the epidermis yet displayed the highest PRE, indicating that mesophyll P allocation does not necessarily increase PRE and suggesting that epidermal P may be more readily recycled than previously thought (Plaxton and Lambers 2015; Hayes et al. 2018).

The PPUE was similar among accessions, including ICC7184 (Figure 2), contrasting with previous observations in Proteaceae from P‐impoverished soils, where preferential mesophyll P allocation increased PPUE (Hayes et al. 2018). Since palisade mesophyll P concentrations and LMA did not differ significantly among accessions (Figure 2), we infer that photosynthetic rate may have reached an optimum at the observed leaf P levels. Several factors may explain why ICC7184 increased epidermal P allocation without reducing mesophyll P: (1) ICC7184 had the smallest leaflet area (Figure S2), potentially increasing the proportion of veins (Sack et al. 2012; Peng et al. 2022); lower P concentration in veins may allow more P to accumulate in the epidermis; (2) despite thinner leaves, LMA remained similar, suggesting increased tissue density, which could facilitate greater P storage in epidermal cells; (3) variation in leaf anatomy, including differences in cellular density or intercellular air spaces, may influence P distribution and mobility within the leaf (John et al. 2017; Harwood et al. 2021); (4) cell‐specific differences in tonoplast Pi affinity may contribute, as the vacuole in mature leaves typically contains 70%–95% of intracellular P_i_ (Bieleski 1973). Future studies examining Pi transporter expression at the cellular level could clarify these mechanisms.

Notably, higher epidermal P concentration was associated with shorter leaf lifespan (Figure 2). ICC13219, with the lowest epidermal‐to‐mesophyll P ratio, exhibited the longest leaf lifespan and the lowest jasmonate concentration in partially senesced leaves (Figure 3). These findings support our second hypothesis: increased allocation of P to the mesophyll P was associated with longer leaf lifespan, and with changes in phytohormone concentrations. Senescence‐associated phytohormones, including ethylene, ABA, jasmonates and SA, are known to promote leaf senescence (Plaxton and Lambers 2015; Zhu et al. 2015; Yu et al. 2021; Cao et al. 2025). Our findings suggest that in chickpea, both jasmonates and salicylic acid were involved in the regulation of leaf lifespan. ICC7184, with a higher epidermal P concentration, exhibited an elevated concentration of OPDA, a JA precursor, and the highest SA concentrations relative to those in the other accessions. Lipid‐derived jasmonates can induce chlorophyll degradation and leaf senescence (He et al. 2002; Lee et al. 2015; Zhu et al. 2015; Tan et al. 2018; Cao et al. 2025), while SA regulates the expression of senescence‐associated genes (SAGs) (Buchanan‐Wollaston et al. 2005; Ji et al. 2016). Together, these results suggest that elevated epidermal P concentrations may trigger SAG upregulation via JA and SA signalling pathways. Further metabolomic analyses across a broader range of accessions would help clarify the regulatory networks linking P allocation, phytohormone metabolism and leaf lifespan.

ABA concentrations were relatively low in ICC7184, consistent with the observed retention of fully senesced leaves on branches (Figure S3). ABA promotes leaf, flower and fruit abscission (Addicott et al. 1968; Lee et al. 2022), suggesting it may act independently of JA and SA in regulating senescence. Integrating single‐cell transcriptomics with cellular P mapping will be crucial for identifying genes that control both cellular P allocation and the expression of these phytohormones.

### 4.3 Tissue [P] Is Negatively Correlated with Tissue [Ca]

Contrary to previous observations that Ca and P are compartmentalised in different cells or organelles, or sequestered in inert, crystalline forms (Boursier and Läuchli 1989; Ding et al. 2018; Hayes et al. 2019a, 2019b; Ye et al. 2021), our study provides the first direct evidence of an antagonistic relationship between the concentrations of Ca and P within the same plant tissues. In photosynthetic mesophyll cells, high Ca concentrations significantly limit increases in P concentration, which may in turn limit photosynthetic capacity. Notably, this negative correlation was observed primarily in mesophyll cells, not in epidermal cells, which helps explain why the highest P concentrations were often found in epidermal cells rather than in mesophyll cells, likely as a strategy to avoid excessive Ca–P precipitation.

We propose that P and Ca coexist in specific compartments within mesophyll cells, based on the observed significant negative correlations (Figure 2). Cytosolic Ca^2+^ cannot serve as a major storage pool, as its concentration is typically maintained around 100 nM (Stael et al. 2012). Chloroplast Ca^2+^ can reach ~15 mM, but further increases would inhibit Calvin–Benson cycle enzymes (Charles and Halliwell 1980; Stael et al. 2012), reducing photosynthetic efficiency, and promoting phosphate precipitation (Kreimer et al. 1987; Johnson et al. 1995). In animal cells, the endoplasmic reticulum (ER) can store Ca^2+^ at ~2 mM through binding to Ca^2+^‐binding proteins, but whether plant ER operates similarly remains unknown. Other organelles, including mitochondria and peroxisomes, typically maintain Ca^2+^ at nanomolar levels (Conn and Gilliham 2010; Stael et al. 2012). Therefore, the relatively high Ca concentrations measured in mesophyll cells likely originate from vacuolar pools rather than the cytoplasm. Similarly, the markedly higher P concentration observed in the epidermis of ICC7184 (Figure 1), which was significantly higher than mesophyll [P], indicates a storage role rather than a reflection of elevated metabolic demand. Mature leaf vacuoles typically occupy more than 80% of the cell volume (Bieleski 1973), and tissue [P] is generally assumed to reflect vacuolar concentrations (Guilherme Pereira et al. 2018; Hayes et al. 2018). Accordingly, the high epidermal [P] is most likely sequestered in vacuoles. Together, these findings suggest that high concentrations of Ca and P coexist within vacuoles, and that their pronounced interactions in mesophyll cells reveal a previously unrecognised regulatory mechanism for maintaining elemental homeostasis and preventing precipitation.

### 4.4 Increased Epidermal P Allocation Was Associated with Elevated Metabolite P Concentrations

Unexpectedly, ICC7184, which allocated more P to its epidermal cells, did not exhibit a significantly higher leaf P_i_ concentration than the other accessions did; instead, it showed higher metabolite P concentrations and lower P_i_ and lipid P proportions (Figure 3). These findings lead to rejection of our hypothesis that increased epidermal P allocation would be associated with a higher P_i_ proportion. The elevated metabolite P concentration in ICC7184 was most likely derived from an increased proportion of metabolite P within mesophyll cells. Metabolite P mainly comprises sugar phosphates, other soluble organic P compounds, and a minor fraction of ATP, all generated through mesophyll‐based photosynthetic and respiratory processes (Hayes et al. 2022; Suriyagoda et al. 2022; Chen et al. 2026). Importantly, the total P concentration in mesophyll tissues of ICC7184 was not higher than that in the other accessions (Table 1), and its leaf‐level P_i_ concentration was similar to—or even lower than—that of other accessions.

The above observations suggest that the elevated metabolic P concentration did not arise from increased P uptake or P_i_ availability. Rather, it reflects a within‐mesophyll reallocation of P, where a larger fraction of P_i_ was converted into metabolically active forms. Under this scenario, mesophyll cells in ICC7184 sustained faster metabolic turnover by drawing P_i_ from soluble storage pools into metabolic P, thereby reducing the internal P_i_ fraction in the mesophyll. To compensate for this reduced mesophyll P_i_ concentration, ICC7184 appeared to redistribute a greater portion of leaf‐level P_i_ to the epidermis, consistent with its higher epidermal P concentration. Collectively, the elevated metabolic P concentration in mesophyll cells and P_i_ accumulation in epidermal cells likely represent coordinated tissue‐level P adjustments, enabling ICC7184 to maintain both enhanced metabolic function and sufficient P_i_ buffering capacity without significantly increasing total leaf P concentrations.

## 5 Conclusion

The present study demonstrates that chickpea accessions exhibit genotypic variation in leaf cellular P allocation under moderate P availability, challenging the monocot–dicot contrast of cellular P distribution. Preferential allocation of P to mesophyll cells was not associated with higher photosynthetic P‐use efficiency or P‐remobilisation efficiency but was, instead, linked to prolonged leaf lifespan and reduced jasmonate concentrations. We further provide evidence of a strong antagonistic correlation between cellular P and Ca concentrations in mesophyll tissues. In addition, increased epidermal P allocation was associated with higher metabolite‐bound P rather than enlarged inorganic P pools. Collectively, these results suggest previously unrecognised links among cellular P allocation, phytohormones, elemental interactions, and leaf lifespan, and provide a framework for understanding plant P‐use strategies.

## Conflicts of Interest

The authors declare no conflicts of interest.

## Supporting information


**Figure S1:** Representative qualitative element maps for five chickpea accessions grown in soil with a moderate phosphorus (P) supply.
**Figure S2:** Representative photos of chickpea plants at six weeks after sowing.
**Figure. S3** Representative photos of chickpea plants at physiological maturity.
**Figure S4:** Changes in (a) chlorophyll concentration and (b) relative chlorophyll content in young fully expanded leaves (S00) and senesced leaves (S50) of five chickpea accessions.
**Figure S5:** Pairwise Spearman correlations between phytohormones and leaf lifespan (LL).
**Table S1:** Detailed information on the five chickpea accessions used in the experiment.
**Table S2:** Total number of cells analysed for X‐ray elemental maps for each accession.

## Data Availability

The data that support the findings of this study are openly available in Github at https://github.com. All data supporting the findings of this study are available at https://github.com/Franke-ovo/CellularP.
