# Supplementary material for: Leaf Tissue‐Specific Phosphorus Allocation Is Linked to Leaf Lifespan in Chickpea Accessions
Source: Plant Cell Environ. 2026 Jun 28;49(11):7781–93. doi: 10.1111/pce.70704 (PMC13646674; doi:10.1111/pce.70704)
Supplement: Supplementary file 1 [file PCE-49-7781-s001.docx]

**Leaf tissue-specific phosphorus allocation is linked to leaf lifespan in chickpea accessions**

Xiaolong Feng^1,2*^, Peta L. Clode^1,3^, Shuyan Li^1,2^, Shu Tong Liu^1^, Jiayin Pang^1,2*^, Gaétan Glauser^4^, Kadambot H. M. Siddique^2^, Hans Lambers^1,2*^

1 School of Biological Sciences, The University of Western Australia, Perth, WA 6009, Australia

2 The UWA Institute of Agriculture, The University of Western Australia, Perth, WA 6009, Australia

3 Centre for Microscopy, Characterisation and Analysis, The University of Western Australia, Perth, WA 6009, Australia

4 Neuchâtel Platform of Analytical Chemistry, University of Neuchâtel, Neuchâtel, 2000, Switzerland

Correspondence:

[xiaolong.feng@research.uwa.edu.au](mailto:xiaolong.feng@research.uwa.edu.au)

[jiayin.pang@uwa.edu.au](mailto:jiayin.pang@uwa.edu.au)

[hans.lambers@uwa.edu.au](mailto:hans.lambers@uwa.edu.au)

**Supplementary table and figure captions :**

**Table. S1** Detailed information on the five chickpea accessions used in the experiment.

**Table. S2** Total number of cells analysed for X-ray elemental maps for each accession.

**Figure. S1** Representative qualitative element maps for five chickpea accessions grown in soil with a moderate phosphorus (P) supply.

**Figure. S2** Representative photos of chickpea plants at six weeks after sowing.

**Figure. S3** Representative photos of chickpea plants at physiological maturity.

**Figure. S4** Changes in (a) chlorophyll concentration and (b) relative chlorophyll content in young fully expanded leaves (S00) and senesced leaves (S50) of five chickpea accessions.

**Figure. S5** Pairwise Spearman correlations between phytohormones and leaf lifespan (LL).

**Table. S1** Detailed information on the five chickpea accessions used in the experiment. PRE, leaf phosphorus-resorption efficiency.

| Accession | Origin | Biological status | Market type | PRE |
| --- | --- | --- | --- | --- |
| ICC 13219 | Iran | Landrace | Desi | High |
| ICC 7184 | Turkey | Landrace | Desi | High |
| ICC 9590 | Egypt | Landrace | Desi | Low |
| ICC 7323 | Russia | Landrace | Pea-shaped | Low |
| ICC 10885 | Ethiopia | Landrace | Kabuli | Low |

**Table. S2** Total number of cells analysed for X-ray elemental maps for each accession.

| Accessions | Cell type (n) | | | | |
| --- | --- | --- | --- | --- | --- |
|  | | Upper epidermis | Palisade mesophyll | Spongy mesophyll | Lower epidermis |
| ICC 13219 | | 134 | 269 | 186 | 151 |
| ICC 7184 | | 125 | 248 | 178 | 141 |
| ICC 9590 | | 127 | 220 | 178 | 139 |
| ICC 7323 | | 141 | 194 | 222 | 159 |
| ICC 10885 | | 109 | 223 | 181 | 124 |

**Figure. S1** Representative qualitative elemental maps of five chickpea accessions grown under moderate phosphorus (P) supply. Maps show the distribution of carbon (used to illustrate tissue structure), calcium, and phosphorus in transverse sections of young fully expanded leaves. Typical cell types used for cellular elemental profiling are labelled. Regions of interest (ROI) were manually defined on the elemental maps using Oxford Instruments AZtec Energy software. Pixels within each ROI were summed and processed to generate quantitative numerical data for determining elemental concentrations in individual cell types and selected tissue regions. Elemental concentrations were measured using cryo-scanning electron microscopy coupled with X-ray microanalysis. Brighter regions within each map indicate relatively higher elemental signal intensity. However, elemental intensities are qualitative and not directly comparable among images because the maps are not shown as element concentration, only corrected X-ray intensities. Concentration data are provided in Figure 1. All elemental maps are displayed using a common white scale. Scale bars = 50 μm.

**Figure. S2** Representative photos of chickpea plants at six weeks after sowing. From left to right: ICC 13219, ICC 7184, ICC 10885, ICC 7323, ICC 9590.

**Figure. S3** Representative photos of chickpea plants at physiological maturity. From left to right: ICC 13219, ICC 7184, ICC 10885, ICC 7323, ICC 9590.

**Figure. S4** Changes in (a) chlorophyll concentration and (b) relative chlorophyll content in young fully expanded leaves (S00) and senesced leaves (S50) of five chickpea accessions. Bars represent means ± SE (n = 4). No significant differences were detected among accessions (Tukey’s test. *P* > 0.05).

**Figure. S5** Pairwise Spearman correlations between phytohormones and leaf lifespan (LL). Phytohormones include salicylic acid (SA), abscisic acid (ABA), jasmonic acid (JA), jasmonoyl-isoleucine (JA-Ile), and 12-oxo-phytodienoic acid (OPDA). Numbers indicate Spearman’s correlation coefficients (ρ). Asterisks indicate statistical significance based on *P*-values (* *P* ≤ 0.05, ** *P* ≤ 0.01).
